# Fundus vascular arcades angle reflects choroidal thickness in highly myopic children and adolescents

**DOI:** 10.1038/s41433-025-03604-9

**Published:** 2025-01-18

**Authors:** Wei Gong, Bo Zhang, Dengji Zhou, Saiguang Ling, Jinliuxing Yang, Jun Chen, Jingjing Wang, Xun Xu, Xiangui He, Wei Gao

**Affiliations:** 1 Shanghai Eye Diseases Prevention &Treatment Center/ Shanghai Eye Hospital, School of Medicine, Tongii University, Shanghai, China; 2 Department of Ophthalmology, Shanghai General Hospital, Shanghai Jiao Tong University School of Medicine, National Clinical Research Center for Eye Diseases, Center of Eye Shanghai Key Laboratory of Ocular Fundus Diseases, Shanghai Engineering Center for Visual Science and Photomedicine, Shanghai, China; 3 EVision technology (Beijing) co. LTD., Beijing, China

**Keywords:** Eye manifestations, Prognostic markers, Refractive errors

## Abstract

**Objectives:**

To identify the role of fundus vascular arcades angle (VAA) in reflecting choroidal thickness (ChT) of highly myopic children and adolescents.

**Methods:**

Participants aged 5 to 18 yrs with high myopia (spherical equivalent, SE ≤ −5.0 D) were enrolled and followed up for one year from the Shanghai Child and Adolescent Large-scale Eye Study. The VAA in the range of one papillary diameter (PD) as well as 2PD away from the central point of optic disc was recognized and measured by artificial intelligence from fundus photographs.

**Results:**

Finally, 277 highly myopic participants were included in the analysis. The mean VAA (1PD) was 128.38 ± 9.56°, and the mean VAA (2PD) was 110.25 ± 11.97°. For those with larger VAA, the choroidal thickness around macula (mChT) or papillary (pChT) was thicker (P for trend < 0.05). After adjusting for age and gender, thinner ChT was independently associated with smaller VAA (*P* < 0.001). For those with more decrease of VAA, the thinning of ChT was more remarkable (*P* < 0.05). In the regression analysis, more change of pChT was independently associated with more change of VAA (*P* < 0.01). After adjusting for other related parameters, 1°change of VAA (1PD) or VAA (2PD) accounted for 0.855 mm or 0.719 mm change of pChT.

**Conclusions:**

Fundus VAA was closely associated with choroidal thickness in highly myopic paediatric population. It could serve as an alternative indicator of choroid thickness in the fundus screening for evaluating the risk of pathological changes of high myopia.

## Introduction

Myopia is a significant public health problem worldwide, which is gaining more attention since its prevalence has sharply increased and the onset age has become younger [1–3]. It has been predicted that, without effective intervention, half of the population would suffer from myopia by 2050 [4]. Especially in East Asia, 39.5% of children have myopia. The prevalence of myopia reaches 80%–90% in high school graduates, of which 10%–20% are with high myopia [5–8].

Myopia can cause irreversible visual impairment through fundus complications like myopic maculopathy [9]. Choroidal thickness (ChT) exhibits a close correlation with the progression of myopic fundus changes. Consequently, the identification of choroid thinning holds considerable significance in the management and control of myopia [10–13]. However, obtaining accurate measurements of choroidal thickness typically requires optical coherence tomography (OCT), which may not be practical or accessible in all settings [14, 15]. In previous studies, several alternative parameters have been used to reflect choroidal thickness, such as fundus tessellation density (FTD), peripapillary atrophy (PPA), and axial length (AL) [16–18]. However, there is still need for more effective and accessible parameters for predicting choroidal thickness.

Since in the progression of myopia, the elongation of AL could change the relative position of fundus tissues, a few studies have used vascular arcades to assess the degree of myopia [19, 20]. However, whether vascular arcades can be used to reflect choroidal thickness was unknown. We have identified a novel parameter, fundus vascular arcades angle (VAA), to describe the relative position of the upper and lower vascular arcades. This study aims to investigate the relationship between VAA and choroidal thickness in individuals with high myopia. If VAA can be validated as a predictor of choroidal thickness, it could offer healthcare professionals a new, non-invasive tool for monitoring and managing myopia, ultimately contributing to better patient outcomes.

## Materials and methods

### Participants

Participants aged 5 to 18 yrs with high myopia (spherical equivalent, SE ≤ −5.0 D) were enrolled in kindergartens, primary schools, and middle schools in Shanghai. The ones with amblyopia and other organic eye diseases (strabismus, congenital cataract, and glaucoma) were excluded. The study protocol was explained to all participants and their guardians, and written informed consent forms were obtained. All participants were treated with the tenets of Declaration of Helsinki. Approval from the Shanghai General Hospital Ethics Committee was obtained (NCT02980445).

### Examinations

All participants underwent a series of examinations, including measurement of height and weight, intraocular pressure (noncontact tonometer, NT-510, Nidek, Japan), AL (IOL master 700, Carl Zeiss Meditec, Germany) and cycloplegic refraction.

For cycloplegia, after a slit lamp examination, one drop of 0.5% proparacaine (Alcaine, Alcon) was given to each eye, followed by two drops of 1% cyclopentolate (Cyclogyl, Alcon), 5 min apart. After approximately 30 min, the absence of light reflex and a pupil diameter larger than 6 mm were considered complete cycloplegia. Otherwise, another drop of cyclopentolate would be given.

The refraction and corneal curvature measurements were acquired by an autorefractor (KR-8900, Topcon, Japan) after inducing cycloplegia. All instruments were calibrated before examination. Three measurements of each eye were obtained in a row, and the procedure would be repeated if the difference between any two records was larger than 0.50 dioptre (D) or 0.05 mm.

The AL was acquired with an IOL Master instrument (Carl Zeiss Meditec, Germany) after calibration. Three measurements of each eye were obtained in a row, and if the difference between any two records was greater than 0.05 mm, the measurement would be repeated.

A swept-source optical coherence tomography (SS-OCT, DRI OCT Triton, Topcon) examination was performed for choroidal thickness. Meanwhile, fundus photographs for the macular areas were acquired with a digital retinal camera in the same SS-OCT system. Segmentation of the different layers on the OCT images was automatically completed by built-in software, followed by manual inspection and correction for misjudgement of the layer borders by an OCT technician. The average ChT around macula (mChT) (6 mm) and papillary (pChT) (4.5 mm) was automatically calculated by the built-in SS-OCT software.

### Recognition of VAA

**Fig. 1:**
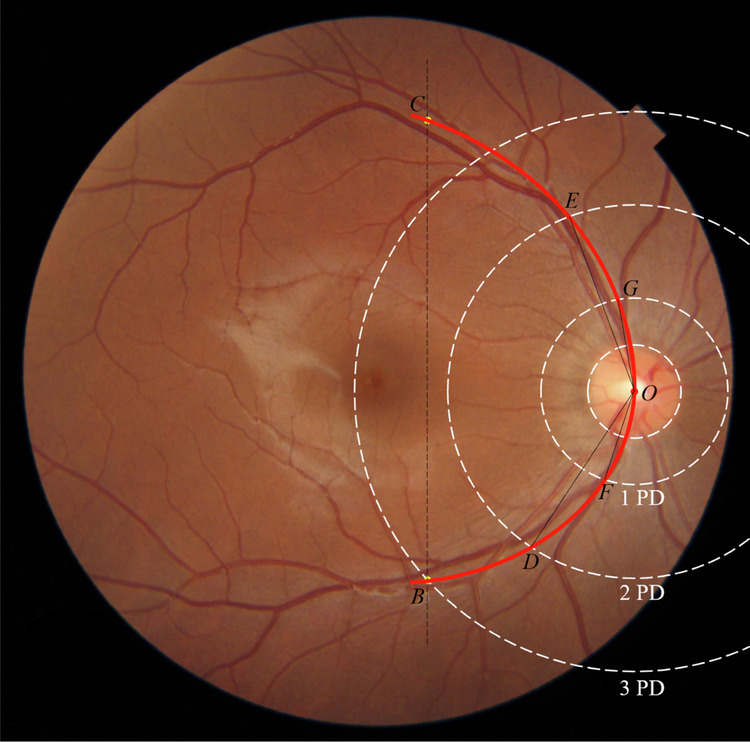
Recognition and measurement of VAA in fundus photograph. Point O is the central point of the optic disc through the optic disc detection. Point B is the midpoint of the intersection point of the thickest artery and vein below the optic disc and the 3PD range. Point C was the centre of the arterio-venous vessels above the optic disc with a vertical line. Two Bezier curves (red) were drawn on the lower and upper sides of the optic disc of OB and OC through point O, and the curve BOC was the fitting curve of the vascular arch. The vascular arch intersects at two points D and E in the 2PD range, and two points F and G in the 1PD range. The angle DOE is VAA (2PD), and the angle FOG is VAA (1PD). PD papillary diameter, VAA vascular arcades angle.

The main method of vascular arch calculation is divided into the following steps (Fig. 1):

1. Image preprocessing: extract the Region of Interest (ROI) area from the fundus photograph, remove black region around the fundus image and non-fundus structures and other invalid areas, and denoise, normalize and enhance the ROI area. The purpose of this step is to weaken the influence of noise in the image, reduce the difference between images, and at the same time highlight the structural features in the fundus image to facilitate subsequent image processing [16].
2. Detection and segmentation of the optic disc: the deep learning target detection method SSD (Single Shot Detection) model is used to locate the optic disc area, and the rectangular bounding box of the optic disc is obtained, and the centre of the bounding box is used as the centre point of the optic disc. Then, the polar coordinate transformation is performed on the area inside the optic disc bounding box with the centre point of the optic disc, the edge detection operator is used to detect the edge of the optic disc in the polar coordinate image, and then the inverse transformation is performed to obtain the edge of the optic disc in the Cartesian coordinate system to realize the segmentation of the optic disc area [21]. Finally, the smallest circumscribed circle was fitted for the segmentation, and the diameter of the circumscribed circle was used as the diameter of the optic disc.
3. Key point positioning: First, locate the central point O of the optic disc through the optic disc detection. Then use the target detection algorithm again to define two points B and C in the image. Point B is the midpoint of the intersection point of the thickest artery and vein below the optic disc and the 3 papillary diameter (PD) range. Point C was the centre of the arterio-venous vessels above the optic disc with a vertical line.
4. Curve fitting of the vascular arch: Two Bezier curves were drawn on the lower and upper sides of the optic disc of OB and OC through point O, and the curve BOC was the fitting curve of the vascular arch. The vascular arch intersects at two points D and E in the 2PD range, and two points F and G in the 1PD range. The angle DOE is the included angle of the 2PD vascular arch, and the angle FOG is the included angle of the 1PD vascular arch.

To verify the results, doctors manually measured the VAA (1PD) and VAA (2PD) respectively, and compared them with the artificial intelligence (AI) measurement results. The results showed that the RMSE (Root Mean Squared Error) of VAA (1PD) and VAA (2PD) measured by AI were 5.89°and 5.65°, respectively, and the relative error was 3.70% and 4.04%. Additionally, the recognition of fundus tessellation density (FTD) and peripapillary atrophy (PPA) was conducted as previously described [16, 22].

### Statistical analyses

Statistical analyses were performed with SPSS (IBM SPSS Statistics, Inc., version 25.0, Chicago, IL, USA). Only data from the right eyes were chosen and included in the final analyses. Continuous variables were described as the mean ± standard deviation, while discrete variables were described as counts (proportions). Spherical equivalent (SE) was calculated as spherical power + 0.5*cylindrical power. High myopia was defined as SE ≤ −5.0D. Normality of data distribution was assessed with Shapiro-Wilk test and the equality of variances was determined with the Levene test. For two-group comparisons, the Student’s t test (equal variances) or unequal variance t test (unequal variances) was applied for normally distributed variables, otherwise the Mann-Whitney U test was performed. The Chi-square test or the Fisher’s exact test was used for the bivariate comparisons of categorical variables. *P*-value < 0.05 was considered statistically significant.

## Results

### General and ophthalmic parameters at baseline and one-year follow-up

Finally, 277 highly myopic participants aged 5–18 yrs were included in the analysis. As shown in Table 1, the mean age at baseline was 13.27 ± 2.90 yrs, and 123 (44.4%) were boys. The mean SE was −+.38.5 ± 2.09 D, the mean AL was 26.66 ± 1.08 mm, the mean VAA (1PD) was 128.38 ± 9.56°, and the mean VAA (2PD) was 110.25 ± 11.97°. In the highly myopic population, VAA (1PD) ranged from 104.75° to 154.21°, and VAA (2PD) ranged from 79.86° to 144.31° (Figure S1A & S1B).

**Table 1:** Systematic and ophthalmic characteristics at baseline and after one-year follow-up.

	Baseline	One-year follow up	Change	*P*-value*
**Age, yrs**	13.27 ± 2.90	14.23 ± 2.99	0.96 ± 0.93	**<** **0.001**
**BMI, kg/m**^**2**^	19.71 ± 4.16	20.48 ± 4.2	0.77 ± 1.42	**<** **0.001**
**AL, mm**	26.66 ± 1.08	26.86 ± 1.09	0.20 ± 0.18	**<** **0.001**
**SE, D**	−8.54 ± 2.09	−8.97 ± 2.12	−0.43 ± 0.46	**<** **0.001**
**mChT (6** **mm), μm**	186.09 ± 50.47	186.59 ± 52.35	0.50 ± 15.23	0.296
**pChT (4.5** **mm), μm**	111.72 ± 36.63	113.41 ± 36.97	1.69 ± 10.94	**0.010**
**FTD (macula), %**	4.86 ± 6.85	5.46 ± 7.52	0.60 ± 0.01	**<** **0.001**
**FTD (disc), %**	6.58 ± 8.82	7.07 ± 8.89	0.49 ± 2.90	**0.012**
**PPA, mm**^**2**^	0.51 ± 0.38	0.56 ± 0.42	0.05 ± 0.15	**<** **0.001**
**VAA (1PD), °**	128.38 ± 9.56	127.82 ± 9.61	−0.67 ± 2.75	**<** **0.001**
**VAA (2PD), °**	110.25 ± 11.97	109.37 ± 12.05	−0.88 ± 4.23	**<** **0.001**

^*^P-value for the comparison between the data at baseline and after one-year follow up.

*BMI* body mass index, *AL* axial length, *SE* spherical equivalent, *mChT* macular choroidal thickness, *pChT* peripapillary choroidal thickness, *FTD* fundus tessellation density, *PPA* peripapillary atrophy, *VAA* vascular arcades angle.

Bold values represent statistical significance *p* < 0.05.

There were significant changes in AL, SE, FTD (macula), FTD (disc), PPA and VAA (1PD), and VAA (2PD) (all *P*-value < 0.05) during the one-year follow-up. The VAA (1PD) changed from 128.38 ± 9.56° to 127.82 ± 9.61° and the VAA (2PD) changed from 110.25 ± 11.97° to 109.37 ± 12.05°. The change of VAA (1PD) ranged from −13.28° to13.76°, and the change of VAA (2PD) ranged from −22.01° to 14.74° (Figure S1C & S1D).

### Correlative factors of VAA

The correlated factors of VAA were analysed (Table 2). In the correlation analysis, both VAA (1PD) and VAA (2PD) were positively correlated with SE, mChT (6 mm), and pChT (4.5 mm), and negatively correlated with AL, PPA, FTD (macula) and FTD (disc) (all *P*-value < 0.05). In the multivariate regression, after adjusting the gender and age, both VAA (1PD) and VAA (2PD) were positively correlated with SE, mChT (6 mm), and pChT (4.5 mm), and negatively correlated with AL, PPA, FTD (macula) and FTD (disc) (all *P*-value < 0.05). The findings suggested the close relationship between VAA and myopia-related parameters.

**Table 2:** Correlative factors of vascular arcades angle.

	VAA (1PD)				VAA (2PD)			
	Correlation analysis		Multivariate regression*		Correlation analysis		Multivariate regression*	
	*r*	*P*-value	Beta (95%CI)	*P*-value	*r*	*P*-value	Beta (95%CI)	*P*-value
**AL**	−0.194	**0.001**	−0.022(−0.034 ~ −0.009)	**0.001**	−0.21	**<** **0.001**	−0.019(−0.028 ~ −0.009)	**<** **0.001**
**SE**	0.142	**0.018**	0.03(0.005 ~ 0.056)	**0.022**	0.161	**0.007**	0.029(0.008 ~ 0.049)	**0.006**
**mChT (6** **mm)**	0.297	**<** **0.001**	1.549(0.944 ~ 2.154)	**<** **0.001**	0.292	**<** **0.001**	1.161(0.676 ~ 1.646)	**<** **0.001**
**pChT (4.5** **mm)**	0.213	**<** **0.001**	0.901(0.459 ~ 1.343)	**<** **0.001**	0.215	**<** **0.001**	0.711(0.358 ~ 1.065)	**<** **0.001**
**PPA**	−0.347	**<** **0.001**	−0.013(−0.017 ~ −0.009)	**<** **0.001**	−0.346	**<** **0.001**	−0.011(−0.014 ~ -0.007)	**<** **0.001**
**FTD (macula)**	−0.179	**0.003**	−0.139(−0.223 ~ −0.055)	**0.001**	−0.18	**0.003**	−0.113(−0.181 ~ −0.046)	**0.001**
**FTD (disc)**	−0.148	**0.014**	−0.155(−0.264 ~ −0.046)	**0.005**	−0.153	**0.011**	−0.136(−0.223 ~ −0.049)	**0.002**

*VAA* vascular arcades angle, *AL* axial length, *SE* spherical equivalent, *mChT* macular choroidal thickness, *pChT* peripapillary choroidal thickness, *FTD* fundus tessellation density, *PPA* peripapillary atrophy.

Bold values represent statistical significance *p* < 0.05.

*age and gender were adjusted in the multivariate regression.

### The choroidal thickness in population with different VAA

**Fig. 2:**
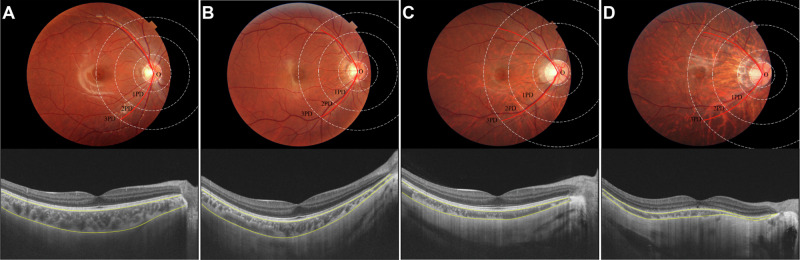
Representative figures of VAA in fundus photographs and corresponding choroidal thickness. Four representative figures of VAA in fundus photographs and corresponding choroidal thickness were shown: (**A**) VAA (1PD) = 146.67°, VAA (2PD) = 134.02°, mChT = 277.56 μm, pChT = 189.84 μm; (**B**) VAA (1PD) = 134.90°, VAA (2PD) = 118.19°, mChT = 194.46 μm, pChT = 162.13 μm; (**C**) VAA (1PD) = 126.58°, VAA (2PD) = 104.77°, mChT = 116.49 μm, pChT = 80.21 μm; (**D**) VAA (1PD) = 114.61°, VAA (2PD) = 90.78°, mChT = 72.01 μm, pChT = 43.87 μm. mChT macular choroidal thickness, pChT peripapillary choroidal thickness, PD papillary diameter, VAA vascular arcades angle.

Representative figures of VAA in fundus photographs and corresponding choroidal thickness were shown in Fig. 2. The population were grouped according to the value of VAA. For the data at baseline, in the groups with larger VAA, the choroidal thickness around macula or papillary were thicker (P for trend < 0.05) (Figure S2). The results showed that individuals with larger VAA had thicker choroidal thickness around the macula or papillary area.

### Correlative factors of the change of VAA

The correlated factors of the change of VAA were analysed (Table S1). In the correlation analysis, both the change of VAA (1PD) and the change of VAA (2PD) were positively correlated with the change of SE and the change of pChT (4.5 mm), and negatively correlated with the change of AL (all *P*-value < 0.05). In the multivariate regression, after adjusting the gender and age, both the change of VAA (1PD) and the change of VAA (2PD) were positively correlated with the change of SE, the change of mChT (6 mm), the change of pChT (4.5 mm), and negatively correlated with the change of AL, the change of FTD (macula) and the change of FTD (disc) (all *P*-value < 0.05). These findings revealed that the change of VAA could reflect the progression of myopia.

### Change of ChT in population with different change of VAA

The population was divided into groups according to change of VAA, and for those with more reduce in VAA (both 1PD and 2PD), the thinning of mChT (6 mm) and pChT (4.5 mm) was more remarkable (*P* < 0.05) (Figure S3). Thus, individuals with a greater reduction in VAA experienced more significant thinning of the choroidal thickness.

### Multiple regression analysis of change of peripapillary choroidal thickness

The multiple regression analysis of the change of pChT (4.5 mm) was conducted (Table 3). After dropping variables with collinearity, more change of pChT (4.5 mm) was independently associated with more change of VAA (1PD), change of VAA (2PD) and change of FTD (disc) (*P*-value < 0.05), but not with change of AL, change of SE or change of PPA. After adjusting for other related parameters, 1°change of VAA (1PD) and VAA (2PD) accounted for 0.855 mm and 0.719 mm change of pChT (4.5 mm). The results suggested change of VAA as a indicator of change of pChT.

**Table 3:** Multiple regression analysis of the change fo peripapillary choroidal thickness.

	Beta	95%CI	Standard Beta	*P*-value	VIF
**Gender, boys**	0.243	−2.327 ~ 2.813	0.011	0.852	1.034
**Age**	−0.569	−1.068 ~ −0.069	−0.153	**0.026**	1.326
**AL change**	−7.521	−16.951 ~ 1.909	−0.129	0.118	1.921
**SE change**	1.143	−2.289 ~ 4.576	0.049	0.512	1.591
**PPA change**	0.026	−8.662 ~ 8.713	0.000	0.995	1.073
**FTD (disc) change**	−0.730	−1.407 ~ −0.053	−0.127	**0.035**	1.021
**VAA (1PD) change**	0.855	0.243 ~ 1.468	0.168	**0.006**	1.062
**Gender, boys**	0.335	−2.228 ~ 2.899	0.015	0.797	1.031
**Age**	−0.555	−1.053 ~ −0.056	−0.149	**0.029**	1.322
**AL change**	−7.776	−17.177 ~ 1.625	−0.133	0.105	1.912
**SE change**	0.843	−2.592 ~ 4.279	0.036	0.629	1.596
**PPA change**	0.129	−8.555 ~ 8.813	0.002	0.977	1.074
**FTD (disc) change**	−0.699	−1.376 ~ −0.022	−0.122	**0.043**	1.022
**VAA (2PD) change**	0.719	0.220 ~ 1.219	0.174	**0.005**	1.067

*VAA* vascular arcades angle, *AL* axial length, *SE* spherical equivalent, *mChT* macular choroidal thickness, *pChT* peripapillary choroidal thickness, *FTD* fundus tessellation density, *PPA* peripapillary atrophy.

Bold values represent statistical significance *p* < 0.05.

## Discussion

The current study raised a new indicator, VAA, to evaluate the degree of myopic fundus changes and choroidal thickness in highly myopic children and adolescents. In the highly myopic population, those with lower VAA were with thinner choroidal thickness. Besides, the change of VAA could reflect the change of choroidal thickness, and had better effect than the change of AL, SE, PPA or FTD when it came to the change of peripapillary choroidal thickness.

The retinal vascular arcades had been described in different methods acting as an index in the assessment of fundus diseases. Two studies identified the relative angle of retinal vascular arcades to describe ocular torsion, which supplemented the traditional way with disc–macula relationship [23, 24]. And in the respect of myopia, Jonas et al. found the association between temporal vascular arcade width and angle and axial length [19]. Another research illustrated that in eyes with an idiopathic macular hole, the vascular arcade has more tendency to diverge on its path temporal to the fovea [20]. The current study gave a more direct relationship between retinal vascular arcades and the degree of myopia as well as myopia-related choroidal thickness.

This study found that individuals with longer AL or more severe myopia presented with a smaller VAA. In axial myopia, AL was correlated with enlargement of the eyeball and caused the changes of fundus structure. According to the clinical observations, ChT in high myopia may continue to reduce with age and lead to more severe myopic fundus diseases [11]. After adjusting for age and gender, VAA was found to be positively associated with mChT and pChT. The elongation of AL could change the relative position of fundus tissues, and the retinal vascular trended to be more crowded. In clinical practice, patients with small VAA are prone to have relatively thin ChT and may be key population of fundus screening.

This study was novel in investigating the correlations between change of ChT and change of VAA, based on fundus photograph and SS-OCT of patients with high myopia, providing a convenient indicator for the monitor of choroidal thickness. SS-OCT was not commonly available in many areas to allow measurement of choroidal thickness. As such, an alternative indicator was necessary to primarily screen population with high risk of choroidal thinning. Fundus photograph became the method of choice. And fundus photograph has become an ideal point of penetration. In previous studies, the progression of PPA, FTD, AL and SE were all proved to be associated with the change of choroidal thickness [11, 25–27]. The current results showed that change of VAA can also be used to reflect the change of choroidal thickness. In the multiple regression analysis, for change of pChT, change of VAA was a more sensitive index than change of SE, AL, PPA or FTD. Thus, VAA could act as an indicator of myopia monitor, since the decrease of VAA suggested the progression of myopic fundus changes.

There were several limitations in our study. First, the current study is a one-year follow up study with only inclusion of Chinese school children, hence the certain patterns of fundus changes during a long time cannot be determined, and future longitudinal studies are needed with inclusion of different ethnicity. Second, the sample size was relatively small, and the cases were rather limited in terms of age, further studies should assess fundus tessellation in wide range of age. Third, the analysis only included data from right eyes to avoid the influence of correlation between the two eyes of the same subjects. However, this caused a partial loss of data and may lead to bias. In subsequent studies, we may comprehensively consider introducing binocular data for validation. Lastly, the effect of VAA on reflecting choroidal thickness in other myopic population apart from high myopia was unknown, needing further research.

In conclusion, this study is novel in reporting that VAA is positively correlated with ChT in highly myopic children and adolescents. Moreover, the change of VAA is a more effective indicator of the change of pChT, compared with the change of several parameters, such as AL, SE, FTD and PPA. Thus, VAA could serve as an indicator of high myopia progressing to pathological myopia and it is essential to use fundus photographs to assess VAA in the management of high myopia so as to identify more high-risk cases.

## Summary

### What was known before

- The elongation of eye could change the relative position of fundus vessels, and a few studies have used vascular arcades to assess the degree of myopia.

### What this study adds

- This study put forward a parameter recognized by artificial intelligence from fundus photographs, fundus vascular arcades angle and verified that it could reflect the value and change of choroidal thickness in highly myopic children and adolescents.
- Fundus vascular arcades angle could serve as an indicator of high myopia progressing to pathological myopia and it is essential to use fundus photographs to assess vascular arcades angle in the management of high myopia so as to identify more high-risk cases.

## Supplementary information

Supplemental material

eye-reporting-checklist

## Data Availability

The datasets generated during and/or analysed during the current study are available from the corresponding author on reasonable request.
