# Supplementary material for: Fundus vascular arcades angle reflects choroidal thickness in highly myopic children and adolescents
Source: Eye (Lond). 2025 Jan 18;39(7):1264–9. doi: 10.1038/s41433-025-03604-9 (PMC12043900; doi:10.1038/s41433-025-03604-9)
Supplement: Supplementary file 1 — Supplemental material [file 41433_2025_3604_MOESM1_ESM.docx]

**Figure S1. Distribution of vascular arcades angle and change of vascular arcades angle**


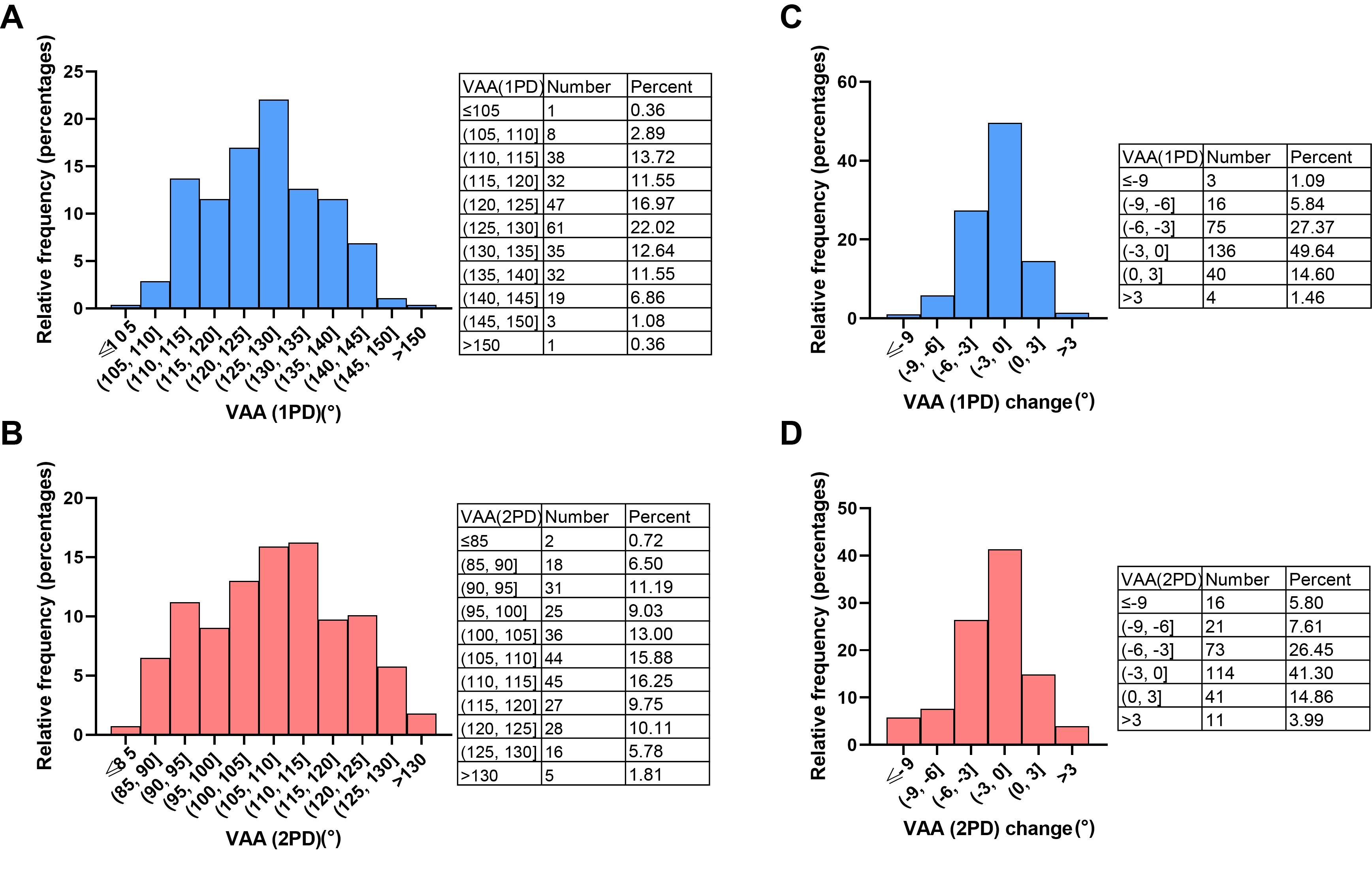


The distribution of vascular arcades angle (1PD) (A), vascular arcades angle (2PD) (B), change of vascular arcades angle (1PD) (C), and change of vascular arcades angle (2PD) (D).

**Figure S2. Choroidal thickness in groups with different** **vascular arcades angle**


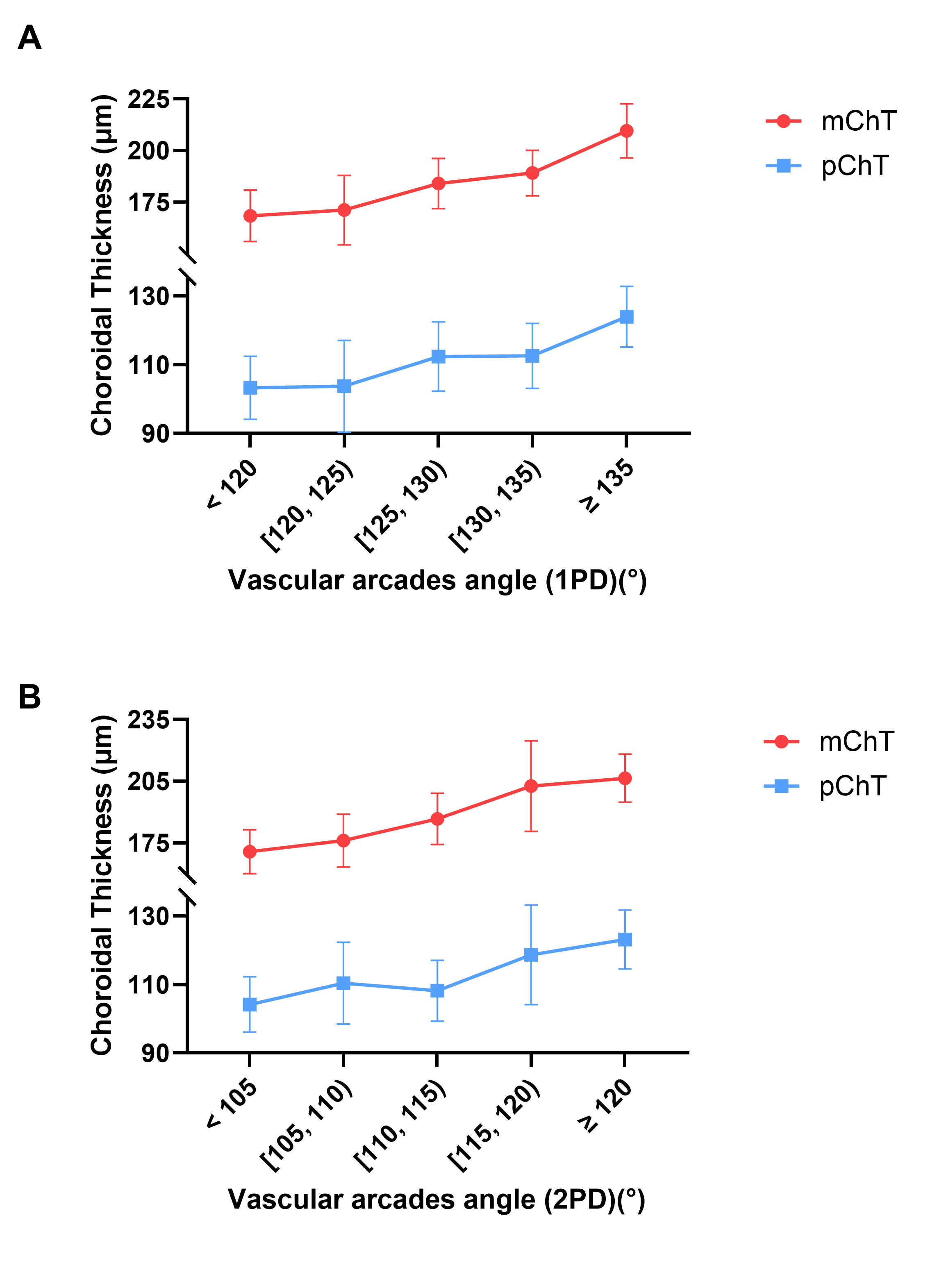


VAA = vascular arcades angle, pChT = peripapillary choroidal thickness, mChT = macular choroidal thickness

**Figure S3. Change of choroidal thickness in groups with different change of vascular arcades angle**

**
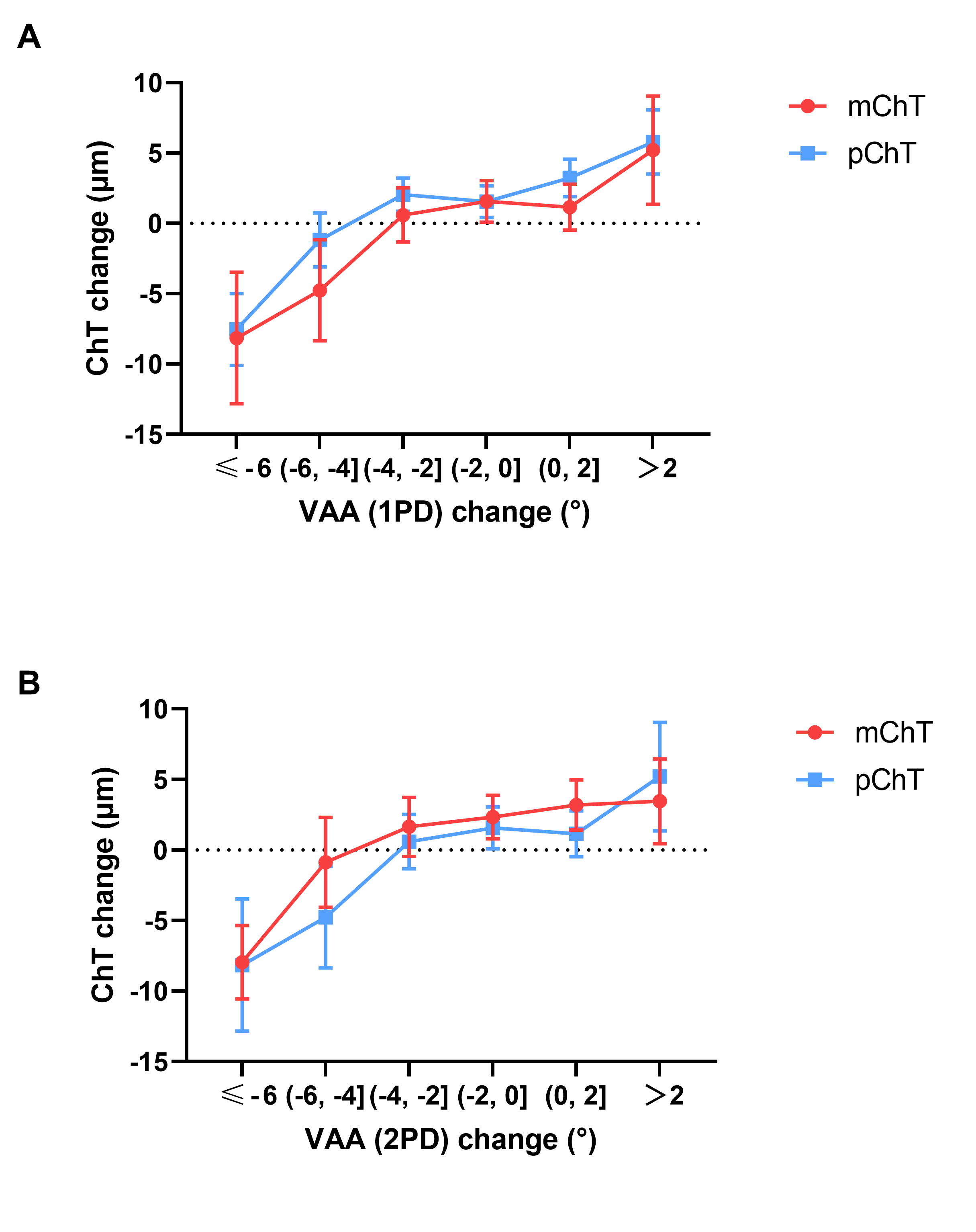
**

VAA = vascular arcades angle, pChT = peripapillary choroidal thickness

**Table S1. Correlative factors of change of vascular arcades angle.**

|  | **VAA(1PD) change** | | | | **VAA(2PD) change** | | | |
| --- | --- | --- | --- | --- | --- | --- | --- | --- |
|  | **Correlation analysis** | | **Multivariate regression*** | | **Correlation analysis** | | **Multivariate regression*** | |
|  | **r** | **P value** | **Beta (95%CI)** | **P value** | **r** | **P value** | **Beta (95%CI)** | **P value** |
| **AL change** | -0.263 | **<0.001** | -0.011(-0.018~-0.004) | **0.002** | -0.270 | **<0.001** | -0.007(-0.011~-0.002) | **0.004** |
| **SE change** | 0.198 | **0.001** | 0.027(0.008~0.045) | **0.006** | 0.207 | **0.001** | 0.016(0.004~0.029) | **0.009** |
| **mChT (6mm) change** | 0.083 | 0.169 | 0.827(0.165~1.489) | **0.014** | 0.070 | 0.246 | 0.568(0.14~0.996) | **0.010** |
| **pChT (4.5mm) change** | 0.171 | **0.005** | 0.846(0.351~1.341) | **0.001** | 0.158 | **0.009** | 0.509(0.194~0.824) | **0.002** |
| **PPA change** | -0.094 | 0.119 | -0.001(-0.008~0.005) | 0.665 | -0.108 | 0.074 | -0.002(-0.007~0.002) | 0.258 |
| **FTD (macula) change** | -0.104 | 0.084 | 0.827(0.165~1.489) | **0.014** | -0.121 | 0.045 | 0.568(0.14~0.996) | **0.010** |
| **FTD (disc) change** | -0.092 | 0.128 | 0.846(0.351~1.341) | **0.001** | -0.082 | 0.175 | 0.509(0.194~0.824) | **0.002** |

VAA = vascular arcades angle, AL = axial length, SE = spherical equivalent, mChT = macular choroidal thickness, pChT = peripapillary choroidal thickness, FTD = fundus tessellation density, PPA = peripapillary atrophy

*age and gender were adjusted in the multivariate regression
